# A novel role of vitronectin in promoting survival of mesenchymal stem cells under serum deprivation stress

**DOI:** 10.1186/s13287-020-01682-y

**Published:** 2020-05-19

**Authors:** Umesh Goyal, Malancha Ta

**Affiliations:** Indian Institute of Science Education and Research (IISER) Kolkata, Mohanpur Campus, Nadia, West Bengal 741246 India

**Keywords:** Vitronectin, MSCs, Cell cycle, Apoptosis, Serum deprivation, Wharton’s jelly

## Abstract

**Background:**

Due to their immunomodulatory and trophic support functions, mesenchymal stem cells (MSCs) are promising in the field of cell-based regenerative medicine. However, MSC survival post-transplantation is challenged by various microenvironment stress factors. Here, we investigated the role of vitronectin (VTN) in the survival strategy of MSCs under serum deprivation stress condition.

**Methods:**

Proliferation kinetics and cell adhesion of MSCs under serum deprivation were determined from population doublings and cell-matrix de-adhesion studies, respectively. mRNA and protein expression levels of VTN were confirmed by qRT-PCR and Western blotting, respectively. Immunofluorescence technique revealed distribution of VTN under serum deprivation stress. siRNA and inhibitor-based studies were performed to confirm the role and regulation of VTN. Apoptosis and cell cycle status of MSCs were assessed using flow cytometric analysis.

**Results:**

Subjecting MSCs to serum deprivation led to significant increase in cell spread area and cell-matrix adhesion. An upregulation of VTN expression was noted with an arrest in G0/G1 phase of cell cycle and no appreciable apoptotic change. Pro-survival PI3kinase pathway inhibition led to further increase in VTN expression with no apoptotic change. siRNA-mediated inhibition of VTN resulted in reversal in G0/G1 cell cycle arrest and a marked increase in apoptosis, suggesting a role of VTN in preventing serum deprivation-induced apoptotic cell death. In addition, p65 knockdown resulted in downregulation of VTN establishing an association between NF-κβ pathway and VTN.

**Conclusions:**

VTN was identified as a survival factor in providing protection from serum deprivation-induced apoptosis in MSCs.

## Background

Mesenchymal stem cells (MSCs) have emerged as promising therapeutic agents in the field of translational medicine. They have proven to be successful in the treatment of a variety of diseases associated with inflammation and tissue damage via their immunomodulatory, anti-inflammatory, and regenerative roles which are mediated in a paracrine manner [1]. However, as against the high expectations, there are obstacles related to MSC applications. As already reported, the transplanted MSCs exhibit low survival rate which is due to harsh microenvironment accompanying a wound, tissue damage, or inflammation in vivo. The harsh microenvironment comprises hypoxia, nutrient deprivation, ischemic condition, inflammation, oxidative stress, etc., and each of these can lead to cell loss [2, 3]. Hence, it is important to overcome the limitations and carefully evaluate in detail the fate and potency of MSCs post-transplantation, in order to target improved efficacy of MSC based therapy.

MSCs have been identified and isolated from a diverse range of adult as well as birth-associated tissues. While MSCs from different sources share some common basic MSC characteristics, they also exhibit some distinct differences in their immunophenotype, immunomodulatory activity, transcriptome, and proteome profile [4]. Though the bone marrow is the most frequently studied and well-characterized source of MSCs, the umbilical cord is a suitable and convenient alternative source, involving non-invasive, hassle-free, and painless collection. Wharton’s jelly (WJ) is the mucoid, connective tissue surrounding the umbilical cord vessels and a rich source of MSCs [5]. Being of neonatal origin, WJ-MSCs have been established to have superior proliferation ability and plasticity with low immunogenicity [6]. Also, there could be significant differences in the way MSCs from different origins respond to microenvironment stress surrounding the cells.

In a recent study of ours, we demonstrated that WJ-MSCs retained greater than 80% viable population under ischemia-like stress condition in vitro [7]. Survival of WJ-MSCs under this harsh, low-nutrient microenvironment was interesting, and hence, the underlying mechanism behind this survival strategy demanded further investigation. Meanwhile, in the same study, we had used the human wound healing RT2 profiler PCR array system and identified a multifunctional glycoprotein, vitronectin (VTN), to be strongly upregulated in ischemic WJ-MSCs. VTN, which is present in large concentrations in serum and ECM, was first described as an adhesive glycoprotein that promoted the attachment and spreading of primary cells on glass. And later, it has been detected in many tissues after exposure to trauma or stress in pathophysiologic settings as a key controller of mammalian tissue repair and remodeling activity [8–10]. Though liver has been shown to be the primary production site, ubiquitous presence of VTN mRNA in several tissues has been detected [11]. In human umbilical vein epithelial cells, VTN has been shown to improve cell survival after radiation injury by attenuating expression of p21 and inhibiting apoptotic cell death [12]. Again, a preferential expression of VTN was reported at the tumor-brain interface in vivo, conferring a survival advantage to the glioma cells [13].

Another report established a novel antiapoptotic function for VTN towards neutrophils through integrin-associated signaling pathways [14]. In the area of stem cells, VTN has been reported to act as an inducer driving cancer stem cell differentiation through an integrin dependent mechanism, while leading to a corresponding downregulation of stem cell maintenance genes in both breast and prostate tissues [15]. In MSCs, both VTN and Col I, as ECM substrates, were shown to support osteogenic differentiation; however, the mechanistic pathways followed were different and substrate dependent [16].

However, not much has been reported on the role of VTN in the survival of MSCs. Motivated by our earlier work, we investigated here the role of VTN in the survival strategy of WJ-MSCs under serum deprivation stress condition. Since an ischemia-like state in vivo involves multiple parameters and is complex to simulate in vitro, in our study here, we considered only nutrient deprivation, which is a key component of ischemia, to investigate the viability of WJ-MSCs and the underlying mechanism.

## Methods

### Cell culture

Human umbilical cords (a total of 9–10) were collected after full-term births (cesarean or vaginal delivery) with informed consent following the guidelines as approved by the Institutional Ethics Committee (IEC) and Institutional Committee for Stem Cell Research and Therapy (IC-SCRT) at IISER, Kolkata, India. WJ-MSCs were isolated from the umbilical cord by explant culture method as described earlier [17]. WJ-MSCs were dissociated with TrypLE Express (Life Technologies, Grand Island, NY, USA), a gentle, animal origin-free recombinant enzyme, and cells were plated at a cell density of 5000 cells/cm^2^ for all experiments. WJ-MSC cultures between passages 4–6 were used for most of the experiments. The MCF7 cell line was a gift from Dr. P. S. Ray (IISER Kolkata, India).

For serum-deprived treatments, WJ-MSCs were first plated in two sets in complete medium comprising of KnockOut DMEM (Dulbecco’s modified Eagle’s medium) supplemented with 2 mM L-glutamine, 10% fetal bovine serum (FBS), and 1X pen strep (all from Life Technologies) for 24 h. Next, cells were subjected to serum deprivation by replacing complete medium with serum-deprived medium (KnockOut DMEM supplemented with 2 mM L-glutamine and 1X pen strep) and incubated for another 48 h. For the control condition, cells were continued to grow in the complete medium for the next 48 h. At the end of 72 h, cells were harvested for the respective experiments. In serum re-addition samples, WJ-MSCs were serum deprived for 36 h and then the medium was replaced by complete medium in which cultures were incubated for another 24 h, following which cells were harvested. For pathway inhibition studies, specific inhibitors or vehicle control were included in the serum deprivation medium and incubated for 48 h. FR180204 as ERK pathway inhibitor, BAY 11-7082 as an inhibitor of NF-κβ pathway, SP600125 as JNK pathway inhibitor, and LY294002 as PI3K pathway inhibitor (all from Sigma Aldrich, Saint Louis, MO, USA,) were added at optimized concentrations of 30 μM, 4 μM, 10 μM, and 20 μM respectively [18–21].

The number of population doublings were calculated using the formula *X* = [log_10_(NH)-log_10_(NI)]/log_10_(2), while the doubling time was obtained by the formula TD=t × log_10_(2)/[log_10_(NH)-log_10_(NI)]. NI is the inoculum cell number, NH is the cell harvest number, and *t* is the time of the culture (in hours).

### Cell spread area determination

To compare the morphology in terms of cell spread area between control and serum-deprived WJ-MSCs, phase-contrast images of WJ-MSCs were captured using Olympus IX81(Olympus, Shinjuku, Tokyo, Japan) inverted microscope at 10X magnification via the micromanager software. Cell area was calculated using the ImageJ (NIH) software. Cell spread area of 50 cells was calculated from each biological sample, and the data were plotted by fitting to the Gaussian equation using the MATLAB 2009 software to get the cell area distribution across the population.

### De-adhesion dynamics

To evaluate the change in adhesion of WJ-MSCs under serum deprivation, time-lapse imaging was done for live cells in the presence of an optimal concentration and volume of TrypLE Express Enzyme (Life Technologies). Live cell imaging was performed using Olympus IX81 (Olympus) inverted microscope via the micromanager software. Images were taken at 10X magnification at an interval of 10 s for a period of 300–500 s as the cells rounded off. Cell spread area was measured across the time period until the cells rounded off and detached from the surface, using the ImageJ (NIH) software. For quantifying the de-adhesion process, cell area was normalized as described previously [22]. The normalized cell area was plotted against time and was fitted in Boltzmann’s equation in the GraphPad Prism 5 (GraphPad, La Jolla, CA, USA) software, and the two time constants were obtained from the graph.

### MTT assay

To assess cell proliferation and viability, MTT assay was performed following the standard protocol. Absorbance was measured at 595 nm using a multimode plate reader (Hidex Chameleon, Lemminkaisenkatu 62, Turku, Finland). Percent viability was calculated with respect to the control after subtracting the background absorbance.

### Cell cycle analysis

To assess cell cycle phase distribution, cells were stained with 50 μg/mL PI (Sigma-Aldrich) containing 100 μg/mL RNase A (Thermo Scientific, LT, Vilnius, Lithuania). Finally, samples were analyzed by BD FACSCalibur flow cytometer, and the fraction of cells in the different phases was assessed using the BD CellQuest software (BD Biosciences, San Jose, CA, USA). A minimum of 10,000 events were acquired per sample.

### Cell viability: annexin-PI staining

To assess the viability of WJ-MSCs following different treatment, floating and adherent populations of cells were harvested and apoptosis assay was performed using Alexa Fluor 488 annexin-V/Dead Cell Apoptosis Kit (Life Technologies, Eugene, OR, USA) as per the manufacturer’s instructions. The percentage of the apoptotic cells was analyzed by flow cytometry (BD FACSCalibur; BD Biosciences). While annexin-V+/PI− population marked the early apoptotic cells, annexin-V+/PI+ labeled the late apoptotic/necrotic population. Ten thousand events were analyzed in each of the samples. Compensation controls were included in every experiment.

### siRNA transfection

After 24–36 h of plating, WJ-MSCs were transfected using Lipofectamine 3000 in Opti-MEM I medium (both from Life Technologies) according to the manufacturer’s protocol. Endonuclease-treated siRNA pools generated against VTN and p65 were used along while mission universal negative control (all purchased from Sigma-Aldrich) at a concentration of 30 nM each. Twenty to 24 h post-transfection, cells were subjected to different treatments for a period of 24–48 h depending on the experiment and then harvested. To test the efficiency of transient transfection method, 1 μg of a GFP plasmid DNA was used. Signal coming from GFP plasmid was visualized using fluorescence microscope. Random fields from two WJ-MSC samples were used to calculate the percentage of transfection efficiency.

### Immunoblotting

Following different experimental treatments, WJ-MSCs were lysed in RIPA buffer containing protease inhibitor cocktail (Sigma-Aldrich) and phosphatase inhibitor (Abcam, Cambridge, UK). Protein concentration was quantified using the Bradford assay. Western blotting was performed as per standard protocols. The primary and secondary antibodies used were as follows: anti-VTN, anti-p53, anti- p65, anti-GAPDH (all from Santa Cruz Biotechnology, Inc. Dallas, TX, USA), and horseradish peroxidase (HRP)-linked anti-mouse IgG (Cell Signaling Technology, Inc., Danvers, MA, USA). Immunoreactivity was detected using chemiluminescent femtoLucent PLUS-HRP (G-Biosciences, Maryland Heights, MO, USA). Chemiluminescent blots were imaged using G:Box (Syngene, Frederick, MD, USA).

### Immunofluorescence

Immunolabeling of control and serum-deprived WJ-MSCs were performed as per standard protocol. WJ-MSCs cultured on glass coverslips, under control and serum-deprived conditions, and were fixed in 4% PFA for 20 min at room temperature. Next, they were permeabilized using 0.1% Triton X-100. Primary antibody incubation was carried out at 4 °C overnight while secondary antibody incubation was performed at room temperature for 1 h. The primary and secondary antibodies used were anti-vitronectin (Santa Cruz Biotechnology, Inc.) and anti-vimentin (Cell Signaling Technology), and goat anti-mouse IgG H&L (Alexa Fluor® 488) pre-adsorbed (Abcam), goat anti-rabbit IgG (H+L) cross-adsorbed secondary antibodies, and Alexa Fluor 568 (Thermo Scientific) respectively. Nucleus was labeled with DAPI (Sigma-Aldrich), following which mounting was carried out with VECTASHIELD antifade mounting medium. Images were acquired in the Zeiss Apotome module microscope using the Zen software.

### RNA isolation and cDNA synthesis

Cells were lysed in Tri-Reagent (Sigma-Aldrich), and total RNA was isolated as per the manufacturer’s protocol. RNA yield was quantified using Nanodrop 2000 spectrophotometer (Thermo Scientific). cDNA synthesis was performed using Verso cDNA Synthesis kit (Thermo Scientific) according to the manufacturer’s instruction.

### Quantitative reverse transcription-polymerase chain reaction

To quantify the change in gene expression under the different treatments, qRT-PCR was performed with specific primers. All the reactions were performed with PowerUp SYBR™ Green Master mix (Applied Biosystems, LT, Vilnius, Lithuania) using the ABI Biosystems Step-onePlus instrument (Applied Biosystems). The StepOnePlus software (version v.2.2; Applied Biosystems) was used to analyze data using the 2-ΔΔCT method. Melting curve analysis was done to confirm single, specific PCR products.

GAPDH was considered as the endogenous control and used to normalize each gene expression level. The primer sequences, NCBI accession numbers, and amplicon sizes are listed in Table 1.

**Table 1:** Primer sequences used for quantitative RT- PCR

Gene	Accession number	Forward primers (5′-3′)	Reverse primers (5′-3′)	Amplicon size (bp)
*GAPDH*	NM_002046.7	GAGTCAACGGATTTGGTCGT	TTGATTTTGGAGGGATCTCG	238
*AURKA*	NM_198433.3	CATGATGCTACCAGAGTCTACC	GAGATCCACCTTCTCATCATGC	336
*CCNA2*	NM_001237.5	CTGCATTTGGCTGTGAACTAC	ACAAACTCTGCTACTTCTGGG	143
*CDKN3*	NM_005192.4	GGCAATACAGACCATCAAGCAA	TGATGATAGATGTGCAGCTAATTTGT	73
*EF2A*	NM_005225.3	CATCCAGCTCATTGCCAAGAAG	GATCCCACCTACGGTCTCCTCA	391
*p21*	NM_000389.5	GAGGCCGGGATGAGTTGGGAGGAG	CAGCCGGCGTTTGGAGTGGTAGAA	221
*p27*	NM_004064.4	CCGGCTAACTCTGAGGACAC	AGAAGAATCGTCGGTTGCAG	120
*p53*	NM_000546.5	GAGCTGAATGAGGCCTTGGA	CTGAGTCAGGCCCTTCTGTCTT	151
*VTN*	NM_000638.4	GGGTCTACTTCTTCAAGGGGAA	AATGAACTGGGGCTGTCTGG	197

### Statistical analysis

Data analysis and graphical representations were performed using the GraphPad Prism 5 software (GraphPad). All data are expressed as mean ± SEM. The analytical methods used were the Student’s two-tailed *t* test, one-way ANOVA, and two-way ANOVA. In multi-group analysis, ANOVA was followed by Tukey’s or Bonferroni’s test. Significance was confirmed at *p* ≤ 0.05 or 0.01 levels. Each experiment was performed with at least 3 independent biological samples.

## Results

### Effect of serum deprivation on morphology, cell spread area, and growth kinetics of WJ-MSCs

**Fig. 1:**
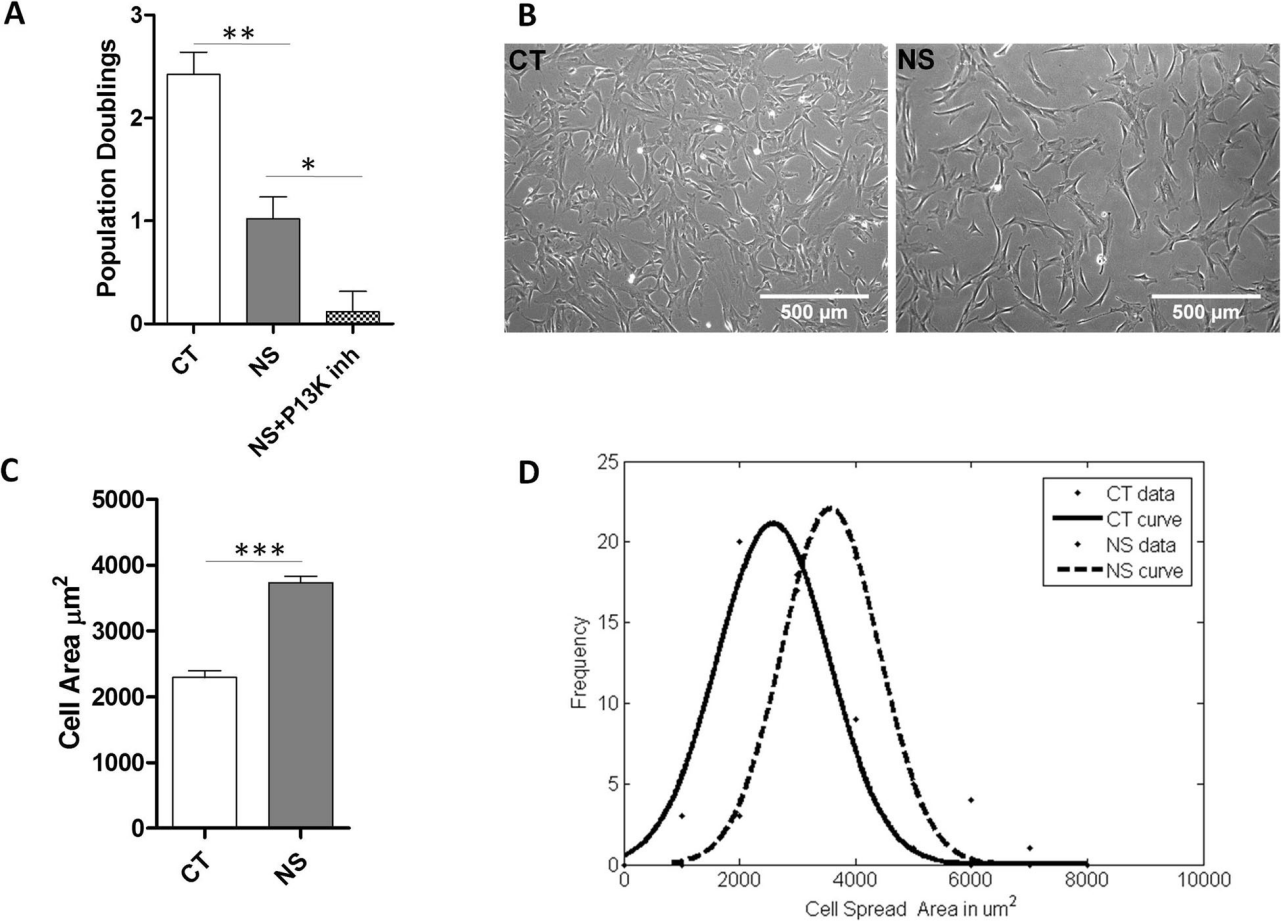
Effect of serum deprivation on growth kinetics and cell spread area of WJ-MSCs. **a** Comparison of population doublings between control, serum-deprived, and PI3Kinase inhibitor (LY294002)-treated WJ-MSCs (*n* = 3). Cultures between passages 4–6 were used. **b** Representative phase contrast images of WJ-MSCs under control and serum-deprived conditions showing morphology are displayed (*n* = 3). **c** Cell area was quantified and plotted for a total of 150 cells from 3 different biological samples and compared. **d** A representative Gaussian distribution plot from 30 cells is presented to show cell area comparison between control and serum-deprived WJ-MSCs. Serum-deprived condition represented as no serum (NS) in the figure. *p < 0.05, **p < 0.01

The effect of serum deprivation on proliferation kinetics of WJ-MSCs was investigated. The number of population doublings of 2.4 ± 0.2 and 1.0 ± 0.2 were noted for control and serum-deprived WJ-MSCs (*p =* 0.0018) (Fig. 1a), respectively, which reflected reduced proliferation rate under serum-deprived condition. The serum-deprived WJ-MSCs also had a longer mean population doubling time of 61.6 ± 7.4 h, while the control WJ-MSCs had mean population doubling time of 28.9 ± 2.9 h (data not shown). Next, the impact of serum deprivation on the morphology of WJ-MSCs was assessed. While control WJ-MSCs cultured in the presence of 10% FBS exhibited a typical fibroblast-like morphology, serum-deprived WJ-MSCs were thinner and longer with a more flattened appearance (Fig. 1b). Cell spread area was measured, and a histogram was plotted (Fig. 1c). A Gaussian distribution of cell area is displayed for control and serum-deprived WJ-MSCs (Fig. 1d).

### Assessment of de-adhesion dynamics and expression of vitronectin and p53 in serum-deprived WJ-MSCs

**Fig. 2:**
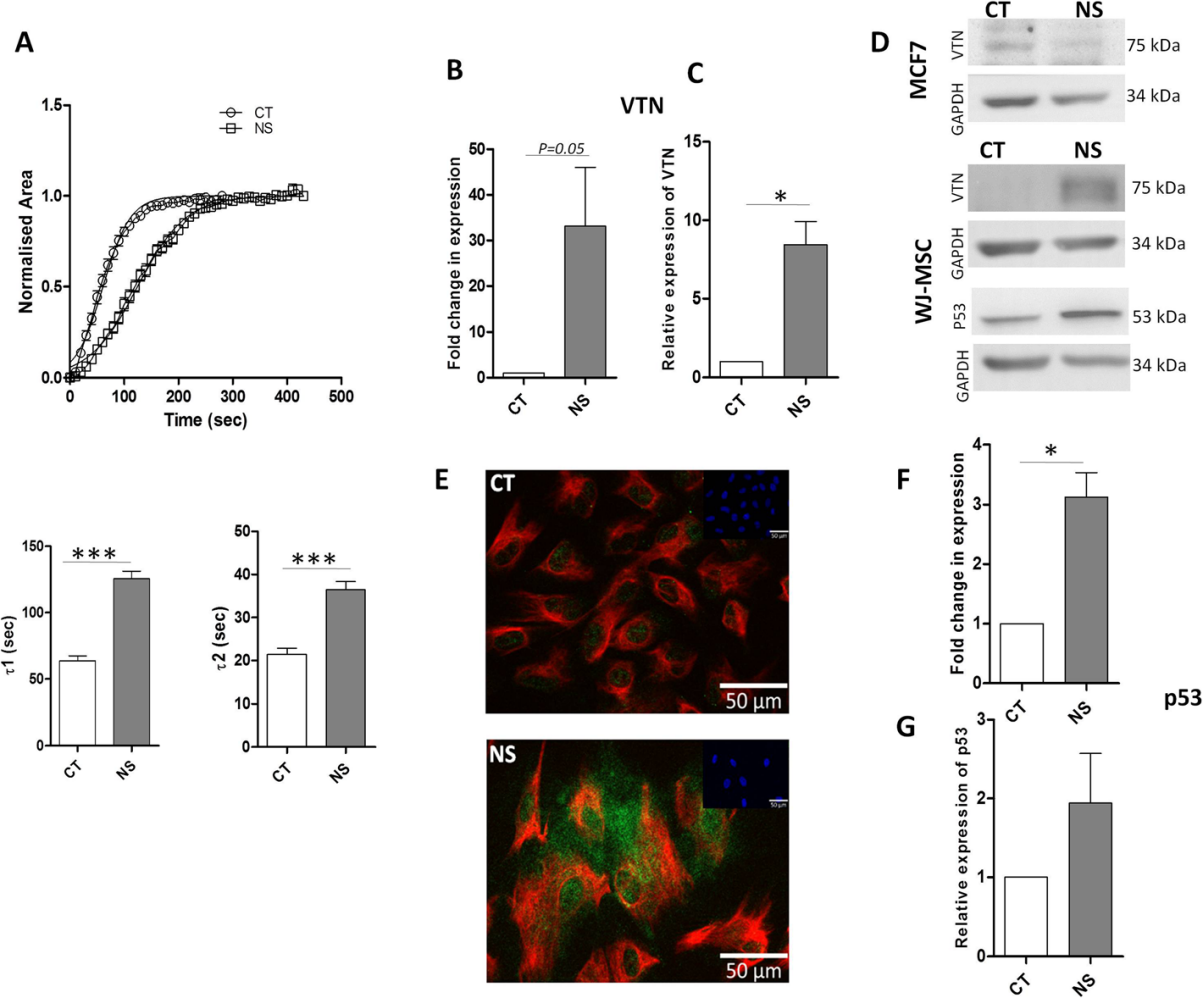
Impact of serum deprivation on de-adhesion dynamics and expression levels of VTN and p53. **a** De-adhesion dynamics of serum-deprived WJ-MSCs. WJ-MSCs were treated with TrypLE and the time taken by the MSCs to contract to a rounded morphology was recorded with the help of time lapse imaging. The normalized area vs. time data were fitted to a Boltzmann sigmoid equation to determine the time constants τ1 and τ2. Delayed de-adhesion leading to greater values for both the time constants were observed. Differences in τ1 and τ2 between control and serum-deprived WJ-MSCs were significant. A total of 45 cells from 3 different biological samples were compared. **b** mRNA expressions of VTN as detected by qRT-PCR (*n* = 5). **c** Band density of protein expression levels of VTN was quantified relative to GAPDH and plotted (*n* = 3). **d** Representative images of Western blots. GAPDH was used as a loading control. MCF7 cells were used as negative control (*n* = 5), as they did not show much increase in expression under serum deprivation. Out of the five sets of experiments performed, two of them showed a small increase in VTN protein expression. **e** The distribution of VTN (green) and vimentin (red) was visualized by immunofluorescence. Insets show nuclei stained with DAPI. Representative images from four independent biological samples are shown (*n* = 4). **f** p53 mRNA was detected by qRT-PCR (*n* = 4). **g** Band density of protein expression levels of p53 was quantified relative to GAPDH and plotted (*n* = 3). Serum-deprived condition represented as no serum (NS) in the figure (*p < 0.05, ***p < 0.0001)

When treated with TrypLE, serum-deprived WJ-MSCs repeatedly demonstrated a brief lag period in detaching from the tissue culture plastic as compared to control WJ-MSCs. We next studied cell contractility and de-adhesion response of control and serum-deprived WJ-MSCs to TrypLE. The detachment response of WJ-MSCs to TrypLE was sigmoidal with three distinct phases, an initial delay period followed by a rapid contraction, and then a plateau. Cell shape change during de-adhesion was quantified, and serum-deprived cells were found to undergo slower detachment as compared to control WJ-MSCs (Fig. 2a).

As VTN has been documented as an adhesive protein, which gets released following exposure to stress and inflammation, we investigated expression of VTN under serum deprivation. A higher expression of VTN, both at mRNA and protein levels as compared to control WJ-MSCs (Fig. 2b–d), was noted. MCF7 human breast cancer cell line was used as control which did not exhibit much of VTN upregulation under serum deprivation (Fig. 2d, Additional file 1: Figure S1). Immunofluorescence staining confirmed the presence and localization of VTN in the control and serum-deprived WJ-MSC cultures. While the control WJ-MSCs expressed VTN in the nucleus, serum deprivation led to a relative increase in expression, extending over to cytoplasm and the ECM as well (Fig. 2e, Additional file 1: Figure S1). Co-staining with vimentin, a mesenchymal marker and an intermediate filament protein showed strong cytoplasmic positivity, under both control and serum-deprived conditions, confirming the mesenchymal nature of these cells (Fig. 2e, Additional file 1: Figure S1).

P53 is a tumor suppressor that acts as a transcription regulator mediating biological effects such as senescence, cell cycle arrest, or apoptosis in response to different forms of stress. There was an induction in p53 expression, as indicated by qRT-PCR and western blotting in serum-deprived WJ-MSCs (Fig. 2d, f, g).

### Effect of PI3kinase pathway inhibition on VTN and p53 expression under serum deprivation stress

**Fig. 3:**
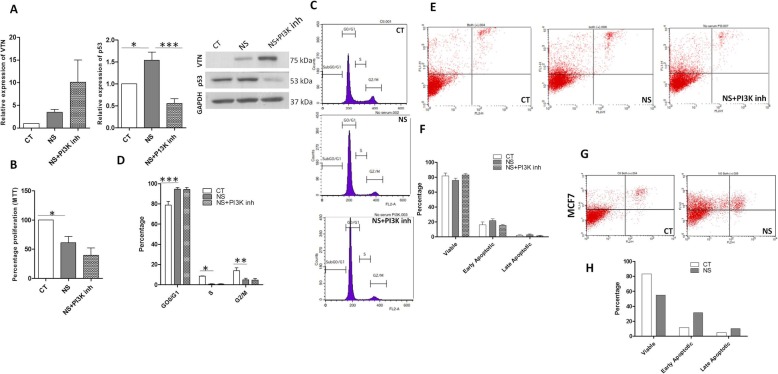
Influence of PI3kinase pathway inhibition on cell proliferation, cell cycle status, and viability of WJ-MSCs under serum deprivation. **a** WJ-MSCs, under serum deprivation, were treated with and without PI3Kinase inhibitor (LY294002) and protein expression levels for VTN and p53 were determined by Western blotting. GAPDH was used to confirm equal loading. Band densities were quantified relative to GAPDH and plotted. Each value represents mean ± SEM of at least four independent biological samples (*n* ≥ 4). **b** MTT assay compared the percentage of metabolically viable cells between WJ-MSCs treated with and without LY294002 under serum deprivation (*n* = 3). **c** Cell cycle analysis of WJ-MSCs, under control and serum-deprived condition, with and without LY294002 treatment. WJ-MSC populations were treated with propidium iodide and DNA content was analyzed by flow cytometry (*n* = 3). **d** Percentages of cells in each phase of the cell cycle are shown by the histograms (*n* = 3). **e** Detection of apoptosis in WJ-MSCs treated with and without LY294002 under serum-deprived condition. WJ-MSCs cultured with 10% FBS were used as control. **f** Comparison of the percentages of viable, early apoptotic, and late apoptotic populations between control and serum-deprived WJ-MSC cultures in the presence and absence of LY294002 as depicted by the histogram. Each bar represents mean ± SEM. Data shown are representative of at least three independent biological samples (푛n=4). **g** Detection of apoptosis in control and serum-deprived MCF7 cells. **h** Comparison of the percentages of viable, early apoptotic, and late apoptotic populations between control and serum-deprived MCF7 cultures. Data shown are representative of two independent experiments. Each bar represents mean. Serum-deprived condition represented as no serum (NS) in the figure (*p < 0.05, **p < 0.01, ***p < 0.0001)

To investigate the underlying molecular mechanism for upregulation of VTN under condition of serum deprivation, we used small molecule inhibitors against certain signaling pathways and tested for VTN expression at both mRNA and protein levels (Additional file 1: Figure S1). On inhibiting PI3 kinase pathway in the serum-deprived WJ-MSCs, VTN was found to be further upregulated both at protein and mRNA levels, though not significant (Fig. 3a, Additional file 1: Figure S1). PI3kinase-Akt pathway is a well-known pro-survival pathway which can regulate various cellular processes such as proliferation, cell growth, and cytoskeletal rearrangement. Studies in the past have shown that Akt can mediate its anti-apoptotic effects in several ways [23].

As p53 plays a vital functional role in response to various stresses as a regulator of cell fate, we also tested the expression of p53 under the serum deprivation treatment with various signaling pathway inhibitors. Interestingly, p53 showed a downregulation by PI3kinase-Akt pathway inhibitor (Fig. 3a, Additional file 1: Figure S1).

### Impact of serum deprivation and PI3kinase pathway inhibition under serum deprivation on cell cycle status and viability of WJ-MSCs

MTT assay data reflected a reduced percentage of metabolically viable cells under serum-deprived condition as compared to control WJ-MSCs (Fig. 3b). This correlated with our population kinetics data indicating growth inhibition under serum deprivation state. Treatment with PI3kinase-Akt pathway inhibitor under serum-deprived state led to further reduction in the metabolically viable cell population, though the difference was not significant (Fig. 3b). A further reduction in number of population doublings also had been noted (0.12 ± 0.19) (*p* = 0.0145) (Fig. 1a). Cell cycle phase analysis using PI staining followed by flow cytometry demonstrated significant differences in G0/G1, S, and G2/M phases of cell cycle between control and serum-deprived WJ-MSCs. An induction in the percentage of G0/G1 phase from 78.8 ± 3.6 to 94.5 ± 1.7 with a concomitant decline in the percentages of S and G2/M phases from 8.4 ± 0.4 and 13.9 ± 2.8 to 0.75 ± 0.1 and 4.7 ± 1.6 (Fig. 3c, d), respectively, was observed. On inhibiting PI3kinase pathway with LY294002 under serum deprivation state, there was no further change in cell cycle profiling as the WJ-MSCs continued to be arrested in G0/G1 cell cycle phase with corresponding reductions in percentages of S and G2/M phase cells, which were comparable to the serum-deprived state (Fig. 3c, d).

Though serum starvation caused cell cycle arrest of WJ-MSCs at G0/G1 phase, no significant level of apoptosis was noted. Cell viability was not found to be significantly affected by serum deprivation in WJ-MSCs (Fig. 3e, f). PI3Kinase inhibitor also did not promote any significant apoptotic changes. There was no difference detected in the viability status (Fig. 3e, f). Interestingly, MCF7 cells showed a significant level of apoptosis within 48 h, under serum deprivation stress (Fig. 3g, h).

### Effect of VTN inhibition on serum-deprived WJ-MSCs

**Fig. 4:**
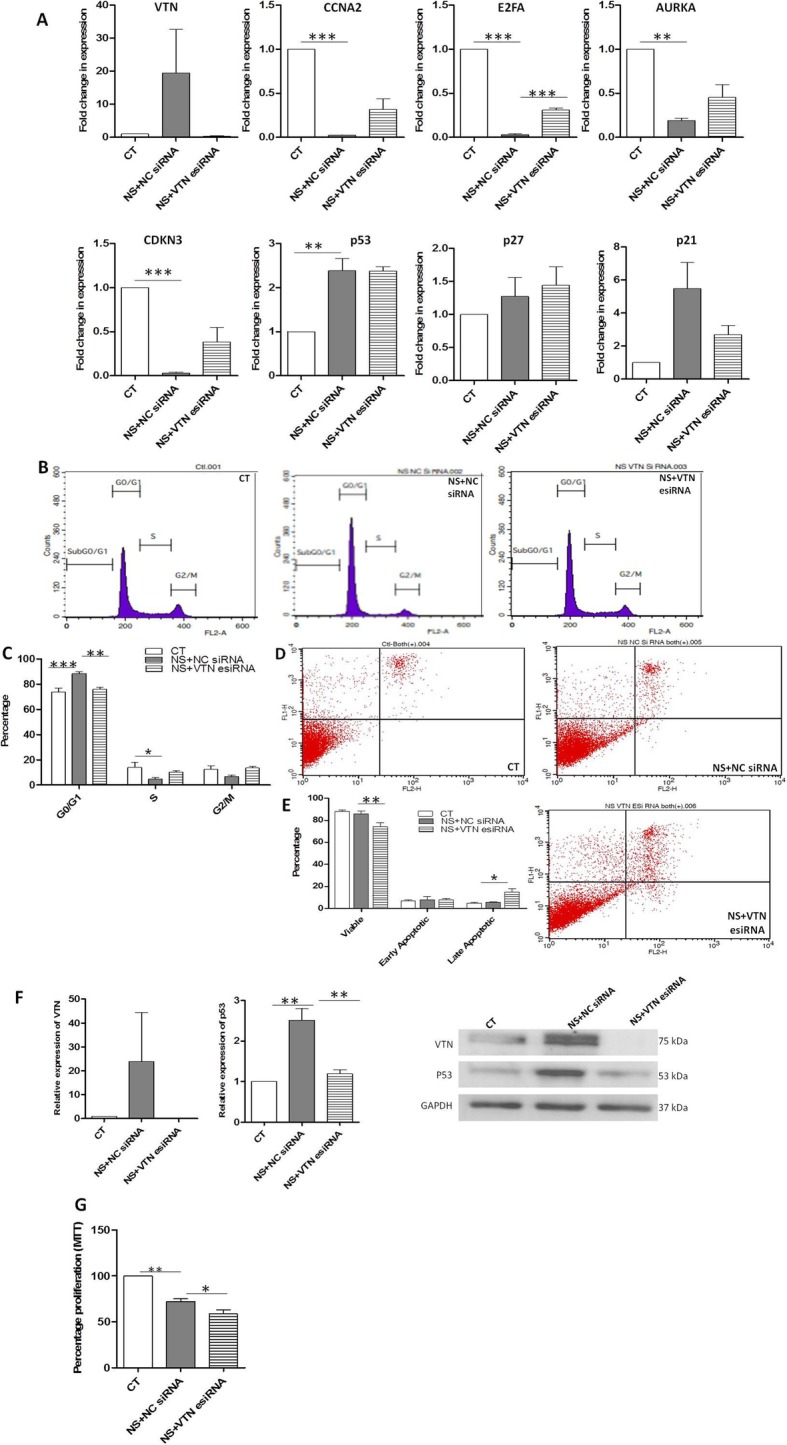
Effect of VTN knockdown on cell cycle status and apoptosis in serum-deprived WJ-MSCs. **a** WJ-MSCs were transfected with a NC siRNA or a VTN-targeted esiRNA for 48 h under serum-deprived state. qRT-PCR analysis in WJ-MSC transfectant cells for VTN, CCNA2, E2FA, AURKA, CDKN3, p53, p27, and p21 are shown (*n* = 3). **b** Cell cycle analysis of WJ-MSCs treated with NC siRNA or VTN esiRNA. **c** Percentages of cells in each phase of the cell cycle are shown by the histograms (*n* = 4). **d** Representative data of apoptosis analysis using flow cytometry are shown. **e** The percentages of viable, early apoptotic, and late apoptotic population of WJ-MSCs transfected with NC siRNA or VTN esiRNA under serum deprivation state are compared and demonstrated using bar graphs (*n* = 3). **f** Knockdown of VTN was achieved in WJ-MSCs following transfection with VTN esiRNA as shown by the Western blot analysis. P53 protein expression too was detected in the VTN knockdown WJ-MSCs. Expression levels were quantitated with respect to GAPDH, which was used as a loading control (*n* = 3). **g** Percentage of metabolically viable population was determined in the NC siRNA vs VTN esiRNA transfected WJ-MSCs by MTT assay. Data shown are representative of three independent biological samples (*n* = 3). Each bar represents mean ± SEM. Serum-deprived condition represented as no serum (NS) in the figure (*p < 0.05, **p < 0.01, ***p < 0.0001)

To examine if VTN was responsible for providing the support for prolonged survival to WJ-MSCs under serum deprivation stress, we next knocked down VTN to test the effect. WJ-MSCs were transfected with VTN esiRNA and exposed to serum deprivation for 48 h. Transfected WJ-MSCs, when assayed for VTN mRNA and protein expression levels, exhibited reduced levels for both mRNA (Fig. 4a) and protein (Fig. 4f, Additional file 1: Figure S1).

The enrichment of cells in G0/G1 phase under serum deprivation was reversed in VTN esiRNA transfected WJ-MSCs, bringing down the percentage from 88.4 ± 1.6 to 75.9 ± 1.6 (*p* ≤ 0.01), as depicted by cell cycle analysis (Fig. 4b, c). There was a corresponding increase in the percentages of cells in S and G2/M phases (Fig. 4b, c). Hence, VTN knockdown removed the cell cycle arrest which was induced by serum deprivation stress. Gene expression of markers associated with the different phases across the cell cycle was studied next. There was reversal in gene expression levels for most of the cell cycle markers studied (Fig. 4a). Thus, our cell cycle phase analysis data is supported by gene expression study. p53 protein level came down significantly as well (Fig. 4f). Its downstream effector, p21, which suppresses Cdk activity required for progression to S phase, was found to increase at mRNA level under serum deprivation and was reversed on knocking down VTN.

On assessing apoptosis, a reduction in the percentage of viable cells from 85.8 ± 3.6 to 76.3 ± 4.0 was noted in WJ-MSCs treated with VTN esiRNA (*p* ≤ 0.01) (Fig. 4d, e). Correspondingly, there was an increase in the late apoptotic population from 5.5 ± 0.7% to 13.7 ± 3.8% in VTN esiRNA treated cells (*p* ≤ 0.05) (Fig. 4d, e). Even our MTT data confirmed the same (Fig. 4g).

### Assessment of VTN knockdown effect on PI3kinase pathway inhibited WJ-MSCs

**Fig. 5:**
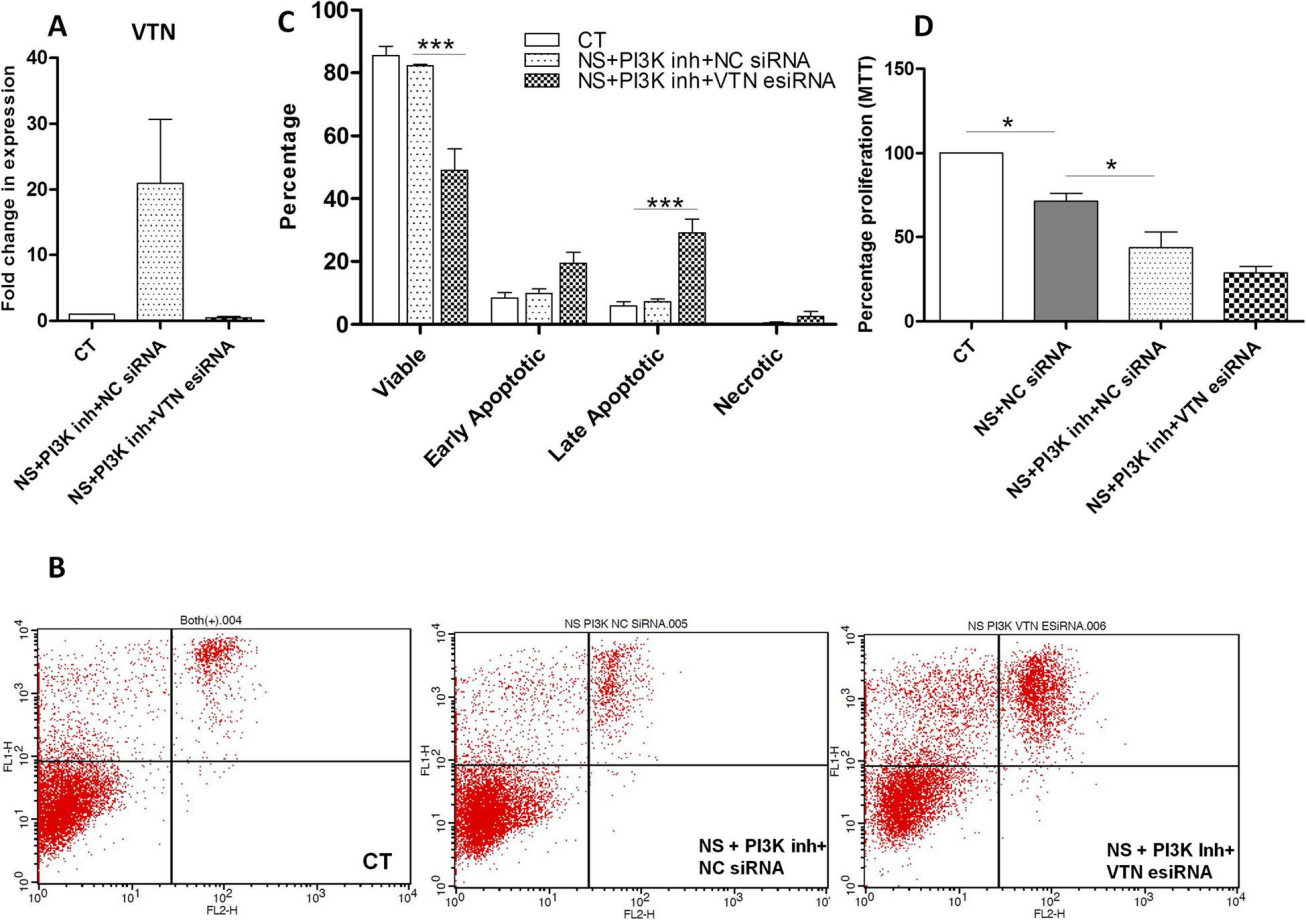
Influence of LY294002 treatment on VTN knocked down WJ-MSCs under serum deprivation. WJ-MSCs transfected with a NC siRNA or VTN-targeted esiRNA were treated with PI3kinase pathway inhibitor, LY294002. **a** qRT-PCR analysis for VTN (*n* = 3). **b** Estimation of apoptosis by annexin V analysis. Representative flow cytometry analysis for detection of apoptosis is displayed. **c** Comparison of the percentages of viable, early apoptotic, and late apoptotic populations between control, NC siRNA-transfected WJ-MSCs treated with LY294002, and VTN esiRNA-transfected WJ-MSCs treated with LY294002 as depicted by the histogram (*n* = 3). **d** MTT assay determined the percentage of metabolically viable population of WJ-MSCs upon LY294002 treatment to the VTN knocked down WJ-MSCs under serum deprivation. Each bar represents mean ± SEM (푛n=3). Serum-deprived condition represented as no serum (NS) in the figure (*p < 0.05, ***p < 0.0001)

WJ-MSCs transfected with VTN esiRNA or NC siRNA were treated with PI3Kinase pathway inhibitor, LY294002, under serum-deprived condition. The treated WJ-MSCs were assayed for VTN mRNA expression and a reduction was noted (Fig. 5a). In the VTN knocked down WJ-MSCs under condition of serum deprivation, treatment with LY294002 led to strong induction in apoptotic signal as compared to NC siRNA treated WJ-MSCs (Fig. 5b). The population of viable cells reduced from 82.2 ± 0.4 to 49.02 ± 6.8 (*p* < 0.001). (Fig. 5c). There was also a marked increase in the percentage of late apoptotic cells which increased from 7.2 ± 0.9 to 29.02 ± 4.4 (*p* < 0.001) (Fig. 5c). Thus, co-inhibition of VTN and PI3kinase signaling pathway strongly promoted apoptosis. MTT assay, which estimates the proliferation and cell viability status in a population of cells, demonstrated a reduction in the viable percentage of cells in the VTN knocked down WJ-MSCs as compared to NC siRNA transfected WJ-MSCs under PI3Kinase pathway inhibition (Fig. 5d).

### NF-κβ pathway acts as a positive regulator of VTN in WJ-MSCs under serum deprivation stress

**Fig. 6:**
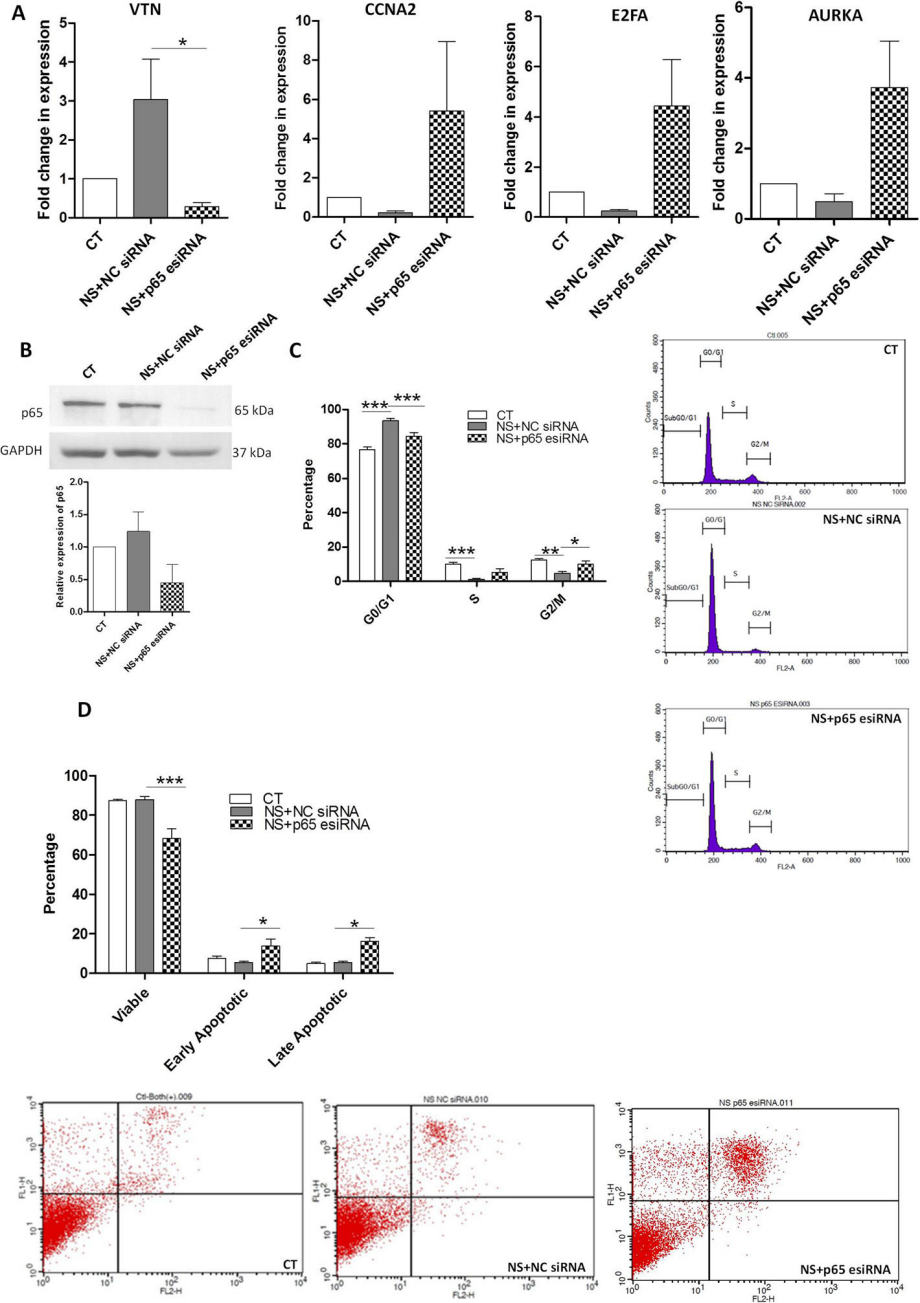
Effect of NF-κβ pathway inhibition on VTN expression, cell cycle status, and cell viability. **a** WJ-MSCs were transfected with NC siRNA or p65 esiRNA and gene expressions of VTN, CCNA2, E2FA, and AURKA was examined using qRT-PCR (*n* = 3). **b** Protein expression of p65 was confirmed to be lowered in the p65 specific knocked down samples by immunoblotting. Band densities were quantified and plotted relative to GAPDH, which was used as a loading control. Blots are representative of three independent biological samples (*n* = 3). **c** Cell cycle phase analysis by flow cytometry. Percentages of cells in each phase of the cell cycle are depicted in the histogram. Bar represents mean ± SEM (*n* = 4). **d** The percentages of viable, early apoptotic, and late apoptotic population of WJ-MSCs transfected with NC siRNA or p65 esiRNA under serum deprivation state are compared and demonstrated using bar graphs. Representative data of apoptosis analysis using flow cytometry are shown. Each bar represents mean ± SEM (*n*=3). Serum-deprived condition represented as no serum (NS) in the figure (*p < 0.05, ***p < 0.0001)

We had observed a decrease in the gene expression for VTN on inhibiting NF-κβ signaling pathway (Additional file 1: Figure S1). This indicated that NF-κβ pathway acted as a positive regulator and was responsible for the induction of VTN under serum deprivation condition in WJ-MSCs. On inhibiting NF-κβ pathway using p65-specific esiRNA (Fig. 6b), there was a downregulation of VTN gene expression (Fig. 6a). We also noted an upregulation in the expression of several cell cycle progression related genes, such as CCNA2, E2FA, and AURKA (Fig. 6a), which were otherwise downregulated under serum-deprived condition. Correspondingly, the cell cycle arrest effect observed at G0/G1 under serum deprivation was partially recovered in the p65 esiRNA-treated samples (Fig. 6c). NF-κβ inhibition increased the apoptotic signal and the viable population percentage decreased from 87.8 ± 1.5 to 68.09 ± 5.05 (*p <* 0.001) with a corresponding increase in early and late apoptotic populations from 5.3 ± 0.7 to 13.89 ± 3.3% and 5.5 ± 0.4 to 16.1 ± 2.03%, respectively (*p* < 0.05 for both) (Fig. 6d).

## Discussion

Stem cell-based therapies have a huge potential to positively impact and revolutionize the conventional treatment of diseases. Stem cells are marked by a property of surviving and supporting endogenous cells during adverse in vivo conditions. However, in reality, tissue regeneration has been compromised by death of transplanted stem cells with most cell death occurring within the first few days post-transplantation. Transplanted cell survival could be challenged by many parameters such as anoikis, mechanical stress, pro-inflammatory factors, oxygen and nutrient deprivation, and the source of MSC itself. Nutrient deprivation is prevalent in the event of tissue injury and wound healing in several disease models such as stroke, myocardial infarction, and critical limb ischemia due to reduction or disruption of arterial blood supply. Hence, understanding MSC response to nutrient deprivation and exploring the underlying mechanism of survival under nutrient deprivation assumes great importance.

There have been contradictory findings regarding the effect of nutrient deprivation on MSCs. Some studies have indicated sensitivity of MSCs to serum deprivation involving alterations in mitochondrial integrity [24, 25]. In contrast, Oskowitz et al. reported a unique phenomenon by which human bone marrow MSCs not only survived for prolonged periods but also secreted pro-survival and angiogenic factors under serum-deprived conditions [26]. Later, another study proposed that ulinastatin was able to promote survival of MSCs under serum deprivation state [27], and similarly, BMP signaling pathway was indicated to be involved in survival of human MSCs in serum-free medium [28]. We also recently showed that WJ-MSCs retained a significant level of viable cells under the harsh ischemia-like conditions in vitro, with a strong upregulation of VTN [7].

On exposing WJ-MSCs to the serum-deprived condition, there was a cell cycle arrest at G0/G1 phase with no detectable apoptosis. Moreover, there was an increase in expression of VTN at both mRNA and protein levels. Our immunofluorescence data revealed redistribution of VTN to the nucleus, cytoplasm, and ECM under serum deprivation. To our knowledge, this is the first demonstration of VTN localization in the nucleus in stem cells and its redistribution under stress condition. VTN has been shown to be an adhesive protein with cell attachment promoting property. In agreement, an increase in cell spread area in conjunction with delayed de-adhesion response to TrypLE treatment were observed too. P53, which is a nodal point for organizing how a cell responds to different types and levels of stress [29], was found to be upregulated. For long PI3 kinases have been implicated in promoting cell survival downstream of extracellular stimuli [23]. Next, on inhibiting PI3kinase pathway under serum deprivation state, we noted a further increased expression trend for VTN with no change in apoptotic signal. On the other hand, p53 protein level went down. An earlier report had shown that p53 mediated transcription was inhibited by pharmacological inhibitors of PI3kinase and pre-treatment with LY294002 altered post-translational modifications and sub-cellular localization of p53 [30]. Moreover, this also indicated that it was VTN, and not p53/p21, which was primarily responsible for suppressing cell cycle progression and possibly essential for the prevention of apoptotic change in the absence of the pro-survival PI3kinase pathway.

To reconfirm the above data, WJ-MSCs were transfected with VTN-specific esiRNA under serum deprivation state, and as expected, there was a reversal in cell cycle arrest. Interestingly, p53 protein level came down significantly as well. Also, the increase in p21, which was observed under serum deprivation state, was reversed by VTN knockdown. This is in contrast to a previous study which reported that VTN inhibited p21 expression and abrogated G1 arrest in endothelial cells after radiation damage [12]. In the same study, VTN was reported to prevent apoptosis. Consistently, in our study, VTN knockdown resulted in a significant reduction in the percentage of viable population with an increase in the late apoptotic population, suggesting that VTN did play a protective role against apoptotic cell death under serum deprivation condition. Furthermore, MCF7 cells, a commonly used human breast cancer cell line of epithelial type, did not exhibit much of VTN induction under serum deprivation and underwent marked apoptosis within 48 h. Though a mesenchymal cell type would have been ideal negative control, most of the commonly available cancer cells are of epithelial origin, which gain mesenchymal nature by EMT. Incidentally, an earlier study had established that IGF-I:IGFBP:VTN complexes enhanced MCF7 breast cell migration and survival, highlighting the interdependence of extracellular matrix and growth factor interactions in biological functions [31]. This further supported our hypothesis.

Potentially, our findings adequately supported that under serum-deprived state, WJ-MSCs underwent cell cycle arrest which in turn helped to protect against apoptosis. This is in sharp contrast to many reports using cancer cell lines where cell cycle arrest and apoptosis have been shown to be induced together [32, 33]. However, in a couple of reports, CDK4/6 inhibition or p21 induction was found to lead cells to cell cycle arrest at G1 which in turn protected them from apoptosis [34, 35]. Interestingly, addition of serum-containing complete growth medium back to serum-deprived WJ-MSCs for a period of 24 h partially reversed the cell cycle arrest effects of serum-starvation (Additional file 1: Figure S1), and this recovery could be time dependent.

Next, as expected, treating VTN knocked down WJ-MSCs with pro-survival PI3kinase inhibitor, LY294002, resulted in even stronger decrease in viable population of WJ-MSCs with a corresponding rise in late apoptotic population. This further stressed the fact that VTN did protect WJ-MSCs from apoptosis induced by serum deprivation stress combined with inhibition of pro-survival PI3kinase pathway. In support, a previous report indicated ECM molecules to promote cell survival and function, particularly in iPSCs, under conditions of ischemia-like stress, while another study proposed enhancement of ECM production as one of the strategies for providing pro-survival cues to improve cartilage tissue yield from iPSCs [36, 37].

Moreover, in our study, inhibition of NF-κβ pathway led to downregulation of VTN gene expression and resulted in blocking the protective effect of VTN, suggesting that the NF-κβ pathway acted as a positive regulator. A previous report had highlighted that NF-κβ mediated αvβ3 integrin-induced rat aorta-derived endothelial cell survival under serum withdrawal [38].

## Conclusion

Hence, all in all, our study has shown VTN as a pro-survival factor which is capable of promoting survival of WJ-MSCs under serum deprivation stress. The importance of VTN is highlighted, and this knowledge will be helpful in redefining and improving therapeutic efficacy of MSCs in wound healing and ischemic diseases. As a future step, it is critical to investigate these findings in animal models where the survival of WJ-MSCs need to be confirmed and compared against other established sources of MSCs under ischemic stress condition, specially, in the context of VTN level of expression and its pro-survival role. Overexpression of VTN could be adopted and tested as a strategy for better survival of MSCs post-transplantation.

## Supplementary information

**Additional file 1: Figure S1.** Effect of different signaling pathway inhibitors on the expression of VTN and p53. WJ-MSCs were treated without or with the indicated inhibitors for 48 h under serum deprivation condition. a VTN mRNA expression was detected using qRT-PCR (*n* = 4). b Protein expression levels of VTN and p53 were detected by Western blotting c, d the band densities of p53 and VTN protein expression were quantified relative to GAPDH, respectively, and plotted (*n* ≥ 4). The difference in p53 protein expression between NS and NS + PI3K inh was found to be significant e Representative immunofluorescence images depicting the expression pattern of VTN (green) and vimentin (red) with insets showing nuclei stained with DAPI f Western blot images of three individual WJ-MSC and MCF7 sample sets demonstrating protein levels of VTN and GAPDH. g Western blot images of three individual WJ-MSC samples demonstrating protein levels of p53, VTN and GAPDH. h In serum re-addition samples, WJ-MSCs were serum deprived for 36 h and then medium was replaced by complete medium in which cultures were incubated for the next 24 h following which cell cycle phase analysis by flow cytometry was performed. Percentages of cells in each phase of the cell cycle are depicted in the histogram. Bar represents mean ± SEM (*n*=3). i Transient transfection effieciency assessment using GFP plasmid. Green fluorescence and the corresponding phase contrast images captured using 10X (should this also be changed to x10? objective are shown. Results are representative of two biological samples. DNA transfection was almost 42%. Serum-deprived condition represented as no serum (NS) in the figure. * represents *p* < 0.05.

## Data Availability

The data sets that support the findings of the current study are available on request from the corresponding author on reasonable request.

## Abbreviations

MSCs: Mesenchymal stem cells
VTN: Vitronectin
NF-κβ: Nuclear factor kappa-light-chain-enhancer of activated B cells
WJ-MSCs: Wharton’s jelly mesenchymal stem cells
ECM: Extracellular matrix
DMEM: Dulbecco’s modified Eagle’s medium
FBS: Fetal bovine serum
PI: Propidium iodide
RIPA: Radioimmunoprecipitation assay
MTT: 3-(4,5-Dimethylthiazol-2-yl)-2,5-diphenyl tetrazolium bromide
CCNA2: Cyclin A2
CDKN3: Cyclin-dependent kinase Inhibitor 3
AURKA: Aurora kinase A
