# Supplementary figures and images for: A novel role of vitronectin in promoting survival of mesenchymal stem cells under serum deprivation stress
Source: Stem Cell Res Ther. 2020 May 19;11:181. doi: 10.1186/s13287-020-01682-y (PMC7238575; doi:10.1186/s13287-020-01682-y)

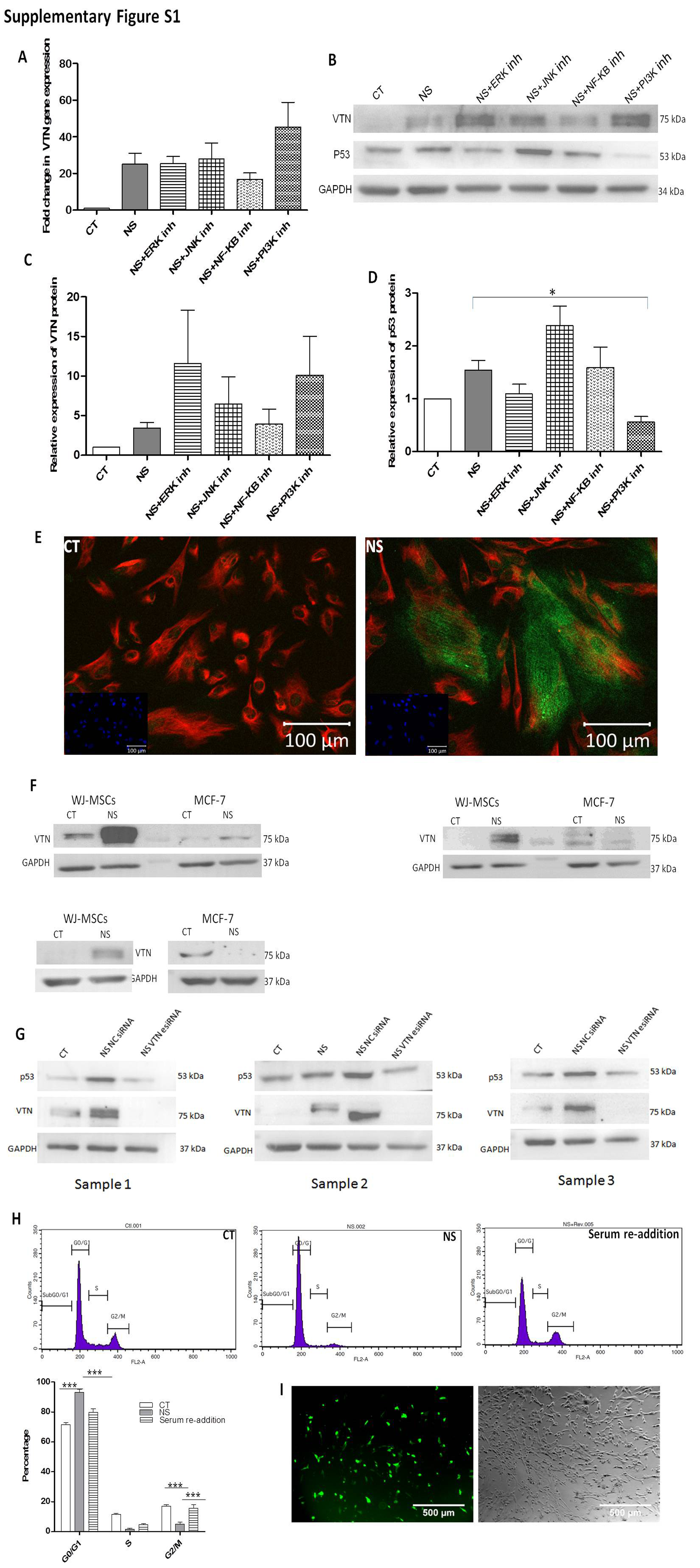

Supplement: Supplementary file 1 — Additional file 1: Figure S1. Effect of different signaling pathway inhibitors on the expression of VTN and p53. WJ-MSCs were treated without or with the indicated inhibitors for 48 h under serum deprivation condition. a VTN mRNA expression was detected using qRT-PCR (n = 4). b Protein expression levels of VTN and p53 were detected by Western blotting c, d the band densities of p53 and VTN protein expression were quantified relative to GAPDH, respectively, and plotted (n ≥ 4). The difference in p53 protein expression between NS and NS + PI3K inh was found to be significant e Representative immunofluorescence images depicting the expression pattern of VTN (green) and vimentin (red) with insets showing nuclei stained with DAPI f Western blot images of three individual WJ-MSC and MCF7 sample sets demonstrating protein levels of VTN and GAPDH. g Western blot images of three individual WJ-MSC samples demonstrating protein levels of p53, VTN and GAPDH. h In serum re-addition samples, WJ-MSCs were serum deprived for 36 h and then medium was replaced by complete medium in which cultures were incubated for the next 24 h following which cell cycle phase analysis by flow cytometry was performed. Percentages of cells in each phase of the cell cycle are depicted in the histogram. Bar represents mean ± SEM (n=3). i Transient transfection effieciency assessment using GFP plasmid. Green fluorescence and the corresponding phase contrast images captured using 10X (should this also be changed to x10? objective are shown. Results are representative of two biological samples. DNA transfection was almost 42%. Serum-deprived condition represented as no serum (NS) in the figure. * represents p < 0.05. [file 13287_2020_1682_MOESM1_ESM.tif]
